# Supplementary figures and images for: Comparing different versions of computer-aided detection products when reading chest X-rays for tuberculosis
Source: PLOS Digit Health. 2022 Jun 14;1(6):e0000067. doi: 10.1371/journal.pdig.0000067 (PMC9931298; doi:10.1371/journal.pdig.0000067)

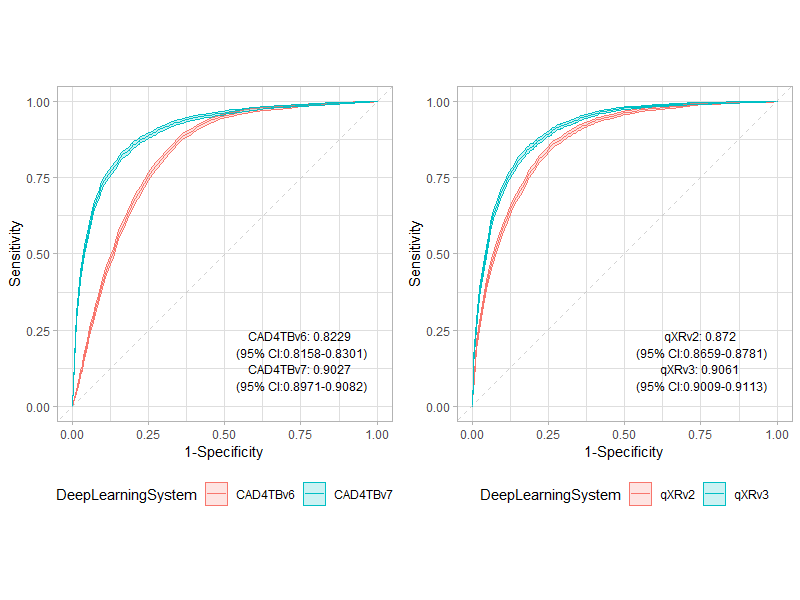

Supplement: S1 Fig — (TIF) [file pdig.0000067.s001.tif]

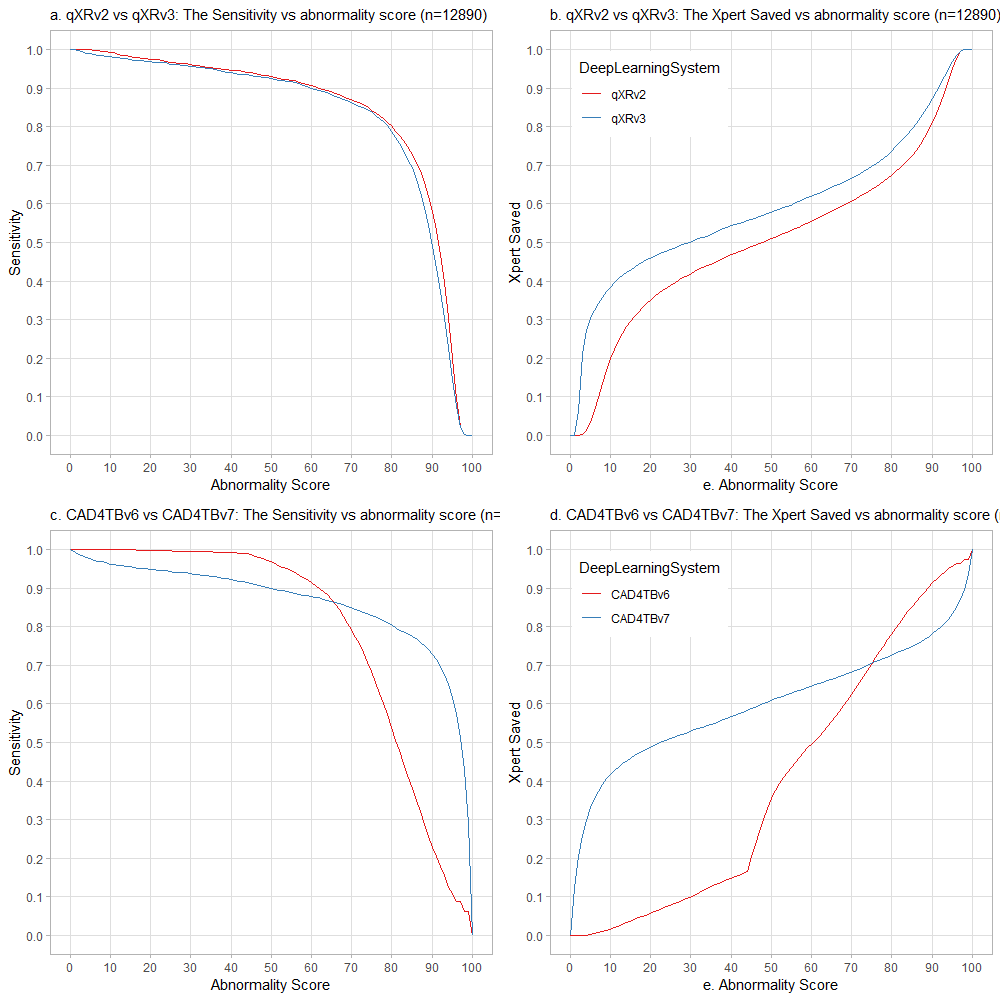

Supplement: S2 Fig — (TIF) [file pdig.0000067.s002.tif]

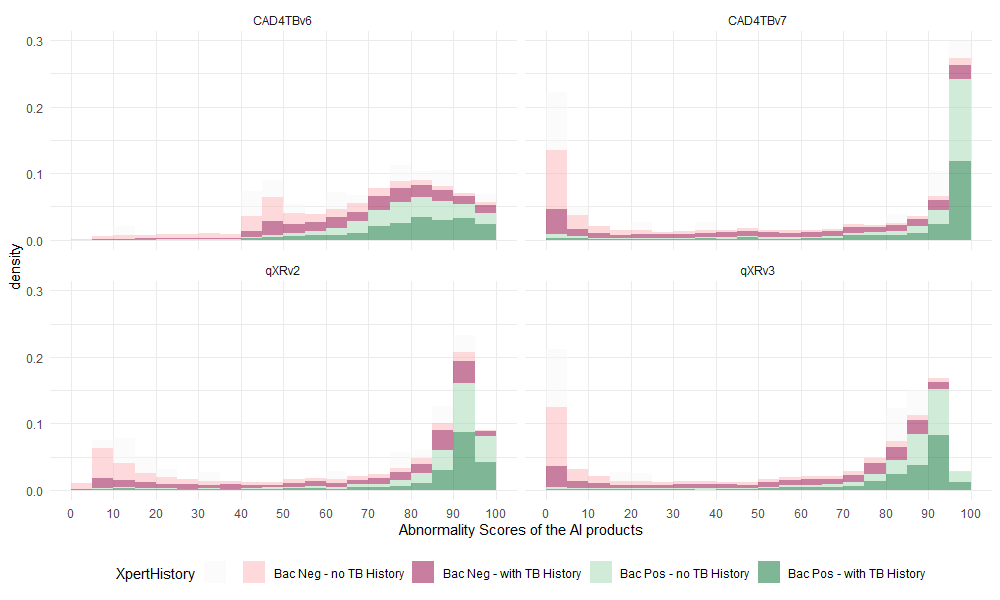

Supplement: S3 Fig — (TIF) [file pdig.0000067.s003.tif]

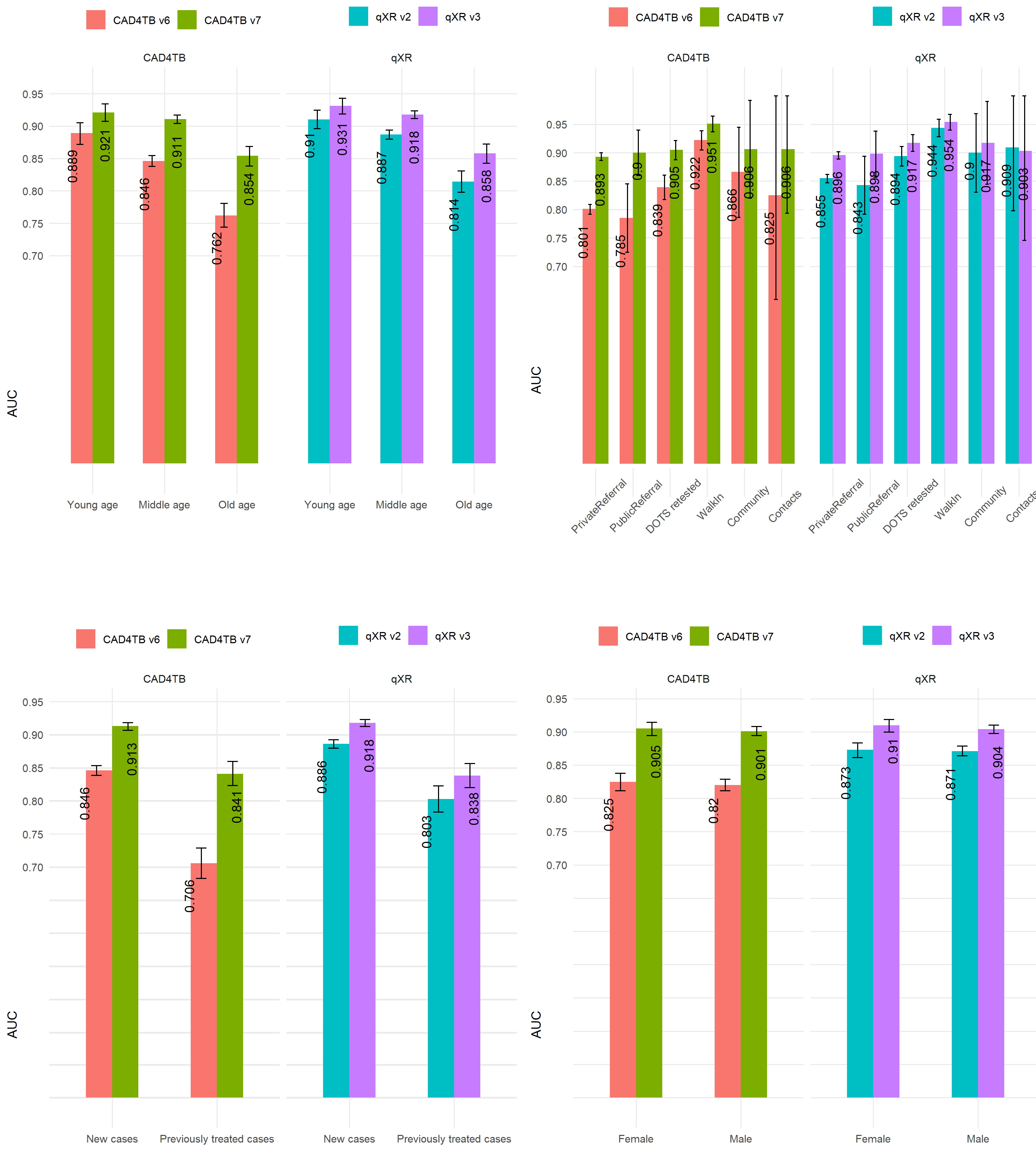

Supplement: S4 Fig — (TIF) [file pdig.0000067.s004.tif]
