# Supplementary material for: Comparing different versions of computer-aided detection products when reading chest X-rays for tuberculosis
Source: PLOS Digit Health. 2022 Jun 14;1(6):e0000067. doi: 10.1371/journal.pdig.0000067 (PMC9931298; doi:10.1371/journal.pdig.0000067)
Supplement: Attachment — Submitted filename: Point by point response to Reviewers comment.docx [file pdig.0000067.s008.docx]

**Rebuttal letter**

___________________________________________________________

We thank the editor and the two reviewers for their comments on our manuscript. Below is our response to each point raised by the academic editor and reviewers. We hope that we satisfyingly addressed them and that the manuscript will be now suited for publication.

Sincerely,

On behalf of all authors,

Zhi Zhen Qin

| **#** | **Reviewer’s comment** | **Response** |
| --- | --- | --- |
| 1 | Reviewer #1: “We therefore compare the performance of two WHO-evaluated CAD product versions with the subsequent versions, using bacteriological evidence as the reference standard. Xpert provided a bacteriological reference standard, confirming the presence (Bac+) or absence (Bac-) of TB.” – could you clarify/confirm if this was for Mycobacterium? | This is a good catch. Yes it is for mycobacterium tuberculosis. We specified this in line 74 |
| 2 | “A dichotomous result (TB-associated abnormalities present or absent) is arrived at by setting a threshold abnormality score, above which the algorithm suggests that TB-associated abnormalities are present, and that the individual should undergo further confirmatory testing.” – Please explain how the threshold abnormality score was set. What technical aspects define a threshold score. | CAD software generates predictions in numeric format between 0 and 1 (or 0-100) which represents the likelihood of having TB or not. The threshold score is nothing but a numeric value selected between 0-1 to dichotomize the CAD prediction into useful binary results (TB or not TB). There are an unlimited number of threshold scores that can be chosen between 0 and 1 and there is no standardized universal threshold score to be used to translate the number to clinical decision. That’s what we study here in this paper, the clinical impact of all possible selection of threshold scores. |
| 3 | “The image was then read by one of three radiologists registered with the Bangladesh Medical and Dental Council. They classified each image as ‘normal’ or ‘abnormal’ (including any abnormality, whether consistent with TB or not).” – what is the radiologists’ interobserver variability?” | Thank you for your question. To clarify, because the image received ONLY one reading from one radiologist – no one CXR read by two radiologists – it is not possible to calculate the kappa or interobserver variability. |
| 4 | “Both products had been reviewed by the WHO Guidelines Development Group and approved for use in TB triage and screening (in individuals ≥15 years) in 2021.” – Why are patients <15 not included based on WHO recommendations for screening with CAD? | If we understand correctly, the question is why WHO recommendation does not include patients under 15. This is because there was no sufficient evidence on the performance of CAD in children under 15 at the time of the guideline development group review. Because we studied the CAD software reviewed and recommended by the GDG at WHO and their latest software versions, we are limited to individual 15 or above for now. The performance CAD of the children under 15 is an important area to research indeed. |
| 5 | “The human radiologists sensitivity was 88.2% (87.2-89.1%) and specificity was 62.8%, (61.8- 190 63.9%) “ – typo for specificity | Thank you for pointing out and apologies. It is corrected now (line 190). |
| 6 | “Matching the sensitivity, all versions had significantly greater specificity 191 than human radiologists except for CAD4TB v6, which was similar.” – Please explain technical aspects that attributed to the improvement in specificity with the newer versions. How was improved specificity defined, the ability for the radiologist to diagnose TB versus other forms of pulmonary diseases? If so, what diseases? What diseases were associated with any false positive or false negative interpretations? | Thank you for your question. To clarify this it is important to set out first that modern CAD software is developed using the AI technique that works by mimicking human brain – neural networks. However, the inner workings of commercial CAD software are challenging to understand for both general audiences and developers, because the nature of neural networks are akin to a black box and the underlying algorithms are the utmost business secret. Therefore, for medical professionals, who will not know any real commercial AI software’s inner workings, confidence in the ability of CAD software to detect TB should be earned by comprehensive and unbiased software evaluations that measure different performance indicators on real-world datasets. We added this in the intro to clarify.    We know that there is a tradeoff between sensitivity and specificity, namely if sensitivity increases the specificity has a tendency to go down. So to measure the changes in one of the two parameters we have to fix the other one. This is the reason why we matched the sensitivity and then compared the specificity of old and new versions to detect TB (not other diseases). But we are not able to explain the technical aspect of what attributed to the improvement of the specificity because of the aforementioned. Our objective as mentioned above is not to explain the inner workings but to conduct an evaluation of the accuracy in an external real world dataset. |
| 7 | Reviewer #2: 1] Overall, the paper is well written and organized.  2] The tables and figures are well organized.  3] The paper provides a statistical data analysis of the previous and current WHO recommended CAD products for TB triaging and screening.  4] The limitations of the study are well stated. | Thank you for your comment |
| 8 | 5] However, the GitHub link provided by the author is not found on the GitHub repository. | <https://github.com/ZZQin/MachineBGD/tree/master/2.0%20Version%20Comparison> |
| 9 | 6] Neural Network is rather a broad term used in the abstract. Can the authors provide a brief paragraph (after introduction) regarding the previous CAD algorithms implemented in CAD4TB (v6) and qXR (v2) compared to the recent CAD powered by AI CAD4TB (v7) and qXR (v3). | Kindly note the elaboration on #6 |
| 10 | This ms addresses the question of automatic TB detection via AI algorithms applied to Xray chest images.  This has potentially significant consequences in terms of pathways of care, their efficacy and cost.  The manuscript lacks a brief intro about the rationale underlying the various AI algorithms compared, their similarity and differences.  In its current version, the study is purely descriptive about algorithm outcomes. Because algorithms have been described elsewhere, it is possible to investigate and discuss at least briefly the sources of difference between algorithms.  Overall, this study is useful, has potentially a significant societal impact but is essentially descriptive and provides no insight about the AI processes at stake. | Kindly note the elaboration on #6 |
| 11 | Please consider also reviewers' comments and my specific comments below:  p4l56 Definition of abnormality score not fully clear | Thank you for the comment, please refer to my response in #2 above. |
| 12 | p5 l88-89 explain why it makes sense to compare score across two different algorithm versions | Thank you for this comment.  There is a lack of understanding that when a CAD software is upgraded, adjustment is also needed otherwise, the sensitivity, specificiation, cost and saving of follow-on testing may or may not change. In some cases, the change is significant. We elaborated in discussion:  “A given threshold score deployed with different versions of the same CAD product will not always be associated with the same sensitivity and Xpert saving, as exemplified by CAD4TB v7 compared to v6.” “For example, using 60 as the threshold score with CAD4TB v6 achieved 92% sensitivity and saved about 43% of Xpert tests. If the software was then updated to v7 and the same threshold used, sensitivity would reduce to 88% and the programme would now save 55% of diagnostic tests.”  However, to make it clearer to reader, we modified and added the following in the introduction:  “More broadly, there is a lack of research in quantifying differences in the programmatic impact between software versions to advise users and TB programmes what and if adjustment is needed when using the software tools that update on a yearly or more rapid basis.” |
| 13 | p6 l108 ref in parentheses unusual and not in ref list | Thank you for the comment. The reference in parentheses on page 6 line 108 (page 6 line 117 in the revised manuscript) refers to the name of the software, version 3.6.0 and location of the library of the statistical software ‘R, v 3.6.0’ (i.e. R Computing, Vienna, Austria). There is no reference intended for the text in the parentheses. R is an open source software that offers a wide variety of statistics-related libraries and provides a favorable environment for statistical computing and design. |
| 14 | Figures are unreadable and simply can not be assessed. Not clear if the pdf file conversion is the culprit or if original figures were of low quality. | Uploaded new Figures with higher resolutions and edited the revised manuscript in track changes accordingly. |
| 15 | Link to github not formatted properly  Actual github repo MAchineBGD does not seem to exist | The link posted on response #8 should solve this now. |
| 16 | I could access repo BGD_AI_for_TB_Detection and it seems neat | Thank you |
